# Endodontic Microsurgery on a Persistent Periapical Lesion

**DOI:** 10.7759/cureus.41250

**Published:** 2023-07-01

**Authors:** Lay Ann Teh

**Affiliations:** 1 Restorative Dentistry, National University of Malaysia, Kuala Lumpur, MYS

**Keywords:** gutta-percha, foreign body reaction, periapical lesion, mineral trioxide aggregates, surgical endodontics

## Abstract

Extrusion of root filling material had been shown to reduce the success of endodontic treatment. This case report describes the management of a patient who reported prolonged, persistent, and increasing pain on an upper root filled central incisor with extruded root filling material.

A 28-year-old female patient came with the chief complaint of pain and tenderness on the upper left central incisor. The pain was mostly triggered by mastication. Upon examination and investigation, the tooth of concern was tooth 21 which was a root treated many years ago. It appeared to have tenderness on percussion and palpation.

Non-surgical root canal retreatment was completed on tooth 21. However, the patient complained of the same pain while biting even after six months post-obturation. Therefore, endodontic microsurgery was performed to remove the root filling material that was extruded and to enucleate the granulomatous lesion around the periapical region of tooth 21. After enucleation, apical root end resection was performed.

Postoperatively, the patient reported comfort and no pain and was able to resume her daily activities. At six months of review, the radiograph showed evidence of complete healing.

This case report captured the importance of endodontic microsurgery as a viable treatment option where nonsurgical root canal retreatment failed to relieve the patient's symptoms.

## Introduction

According to the European Society of Endodontology guidelines 2006, findings that indicate favorable outcomes are the absence of pain, swelling, or other discomforts. Evaluation of clinical success includes the absence of a sinus tract and the tooth remains functional. Radiographically, a good indicator of success is when there is resolution of apical radiolucency and evidence of a re-establishment of the lamina dura.

The success of nonsurgical root canal treatment is defined as the absence of pain, swelling, and other symptoms, no sinus tract, no loss of function, and radiological evidence of normal periodontal ligament. Another good indicator of a favorable prognosis is the resolution of apical radiolucency and evidence of a re-establishment of the lamina dura. One of the clinical factors which influence the success of root canal treatment robustly is the apical extent of the root filling material. Extrusion of root filling material beyond the apical terminus into surrounding tissue may result in delayed healing or treatment failure due to foreign body reaction [1].

The incidence of apical periodontitis in teeth with root canal obturation at 1 mm beyond the apex is 80%, a 16-fold increase when cone beam computed tomography (CBCT) was used as the radiographic imaging of choice [2]. A root filling material that extends beyond can indicate contamination with dentin extrusion, irrigation solution, and filling materials, which act as irritants in the periapical tissue [3]. The cause of extrusion of root filling is due to loss of apical stop during cleaning and shaping and lack of consistent length control. One of the most significant complications related to or that occurs as a consequence of apical extrusion during root canal procedures is interappointment flare-ups and postoperative pain which is an undesirable occurrence both for the patient and the practitioner.

Oftentimes, extrusion of root filling may not pose any severe symptoms and monitoring will suffice. However, in cases where the extrusion of root filling material causes a flare-up and moderate to severe discomfort, endodontic microsurgery is indicated to enucleate the granulation tissue and the foreign body material.

A root filling material that releases components such as formaldehyde and eugenol may elicit a robust inflammatory reaction and may need prompt surgical intervention before these substances cause harm to adjacent vital structures such as nerve bundles. Depending on the condition that the patient presents such as paraesthesia, pain, and swelling, surgical intervention is a predictable management that has been shown to alleviate the symptoms. The success of endodontic microsurgery using modern techniques has been shown to be 94% [4].

In this case report, the outcome of the endodontic treatment was unfavorable as it does not adhere to the criteria of success described above. Although the obturation appeared homogenous and without voids, the patient still suffered from pain. Hence, endodontic microsurgery to manage post-treatment apical periodontitis specifically caused by foreign material extrusion is discussed in detail. Endodontic microsurgery has substantially transformed over the years to make the procedure more precise, detailed, and predictable. Modern techniques in endodontic microsurgery include the use of ultrasonic tips, high-power magnification, and illumination. The instruments such as micro-mirrors are designed to manipulate smaller areas without excessive tissue damage. Instead of amalgam, biocompatible materials are advocated to encourage better healing of the periapical tissues.

## Case presentation

A 28-year-old Chinese female complained of pain and tenderness in the upper left front tooth. The pain was moderate with a Visual Analogue Scale (VAS) of 5/10. A VAS is one of the pain rating scales in epidemiologic and clinical research to measure the intensity or frequency of various symptoms. The pain was triggered by mastication but did not disturb the patient's sleep. Hence, no analgesics were taken. At the age of nine years old, she fell onto the floor and fractured her front teeth. No dental treatment was sought. Six years later at the age of 15 years old, she noticed brownish discoloration on her upper left front tooth. Tooth 21 was root treated by a dental general practitioner. In the course of the next 15 years, she had multiple episodes of pain and swelling during which she saw other three dentists who prescribed antibiotics and analgesics. The symptoms subsided only to recur again every few months.

**Figure 1:**
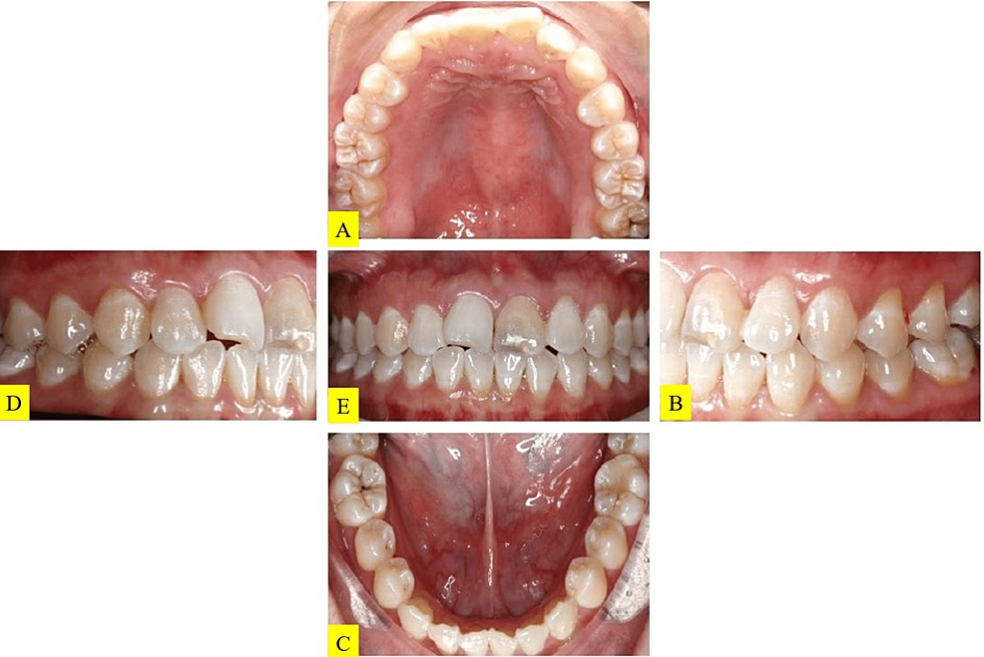
Preoperative intraoral photographs (A) upper occlusal view. (B) left lateral view. (C) lower occlusal view. (D) right lateral view. (E) frontal view

The patient is medically fit and healthy. She is an irregular dental attendee, brushed twice daily with fluoridated toothpaste, and did not use floss or mouth rinse. She is a factory worker and did not consume alcohol or smoke. She did not have any parafunctional habits. There were no abnormalities extraorally and intraorally except for localized gingival inflammation at the lower anterior teeth. Oral hygiene was fair with localized supragingival calculus on the lower lingual anterior teeth. All her teeth were sound with the exception of tooth 46 which had a composite restoration on the occlusal. The patient's overall dentition was recorded in Figure 1.

**Figure 2:**
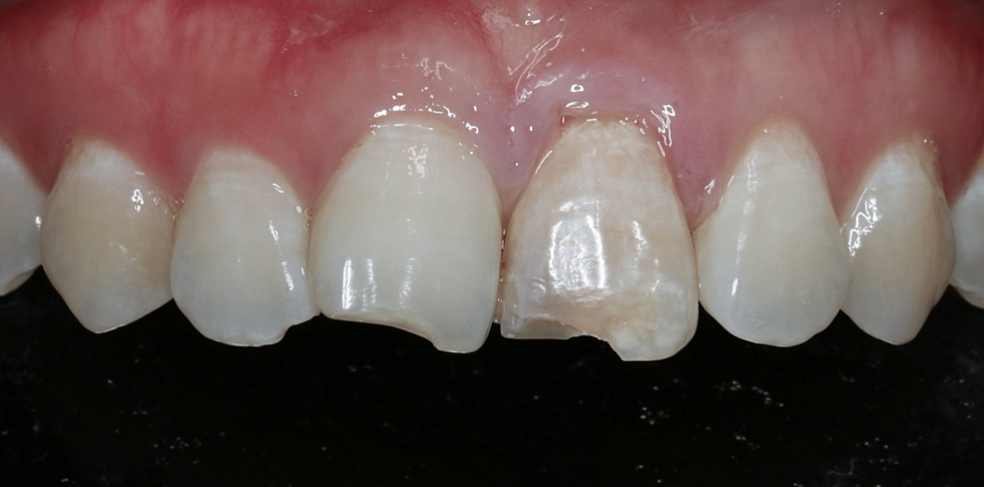
Intraoral picture of tooth 21 (area of complaint)

At the site of complaint tooth 21, the incisal edge was fractured and the crown appeared brownish-grey. The tooth was tender to percussion and palpation at a VAS score of 5/10 and 4/10, respectively. It had grade 1 mobility and periodontal probing depth circumferentially of 2 mm (Figure 2).

**Figure 3:**
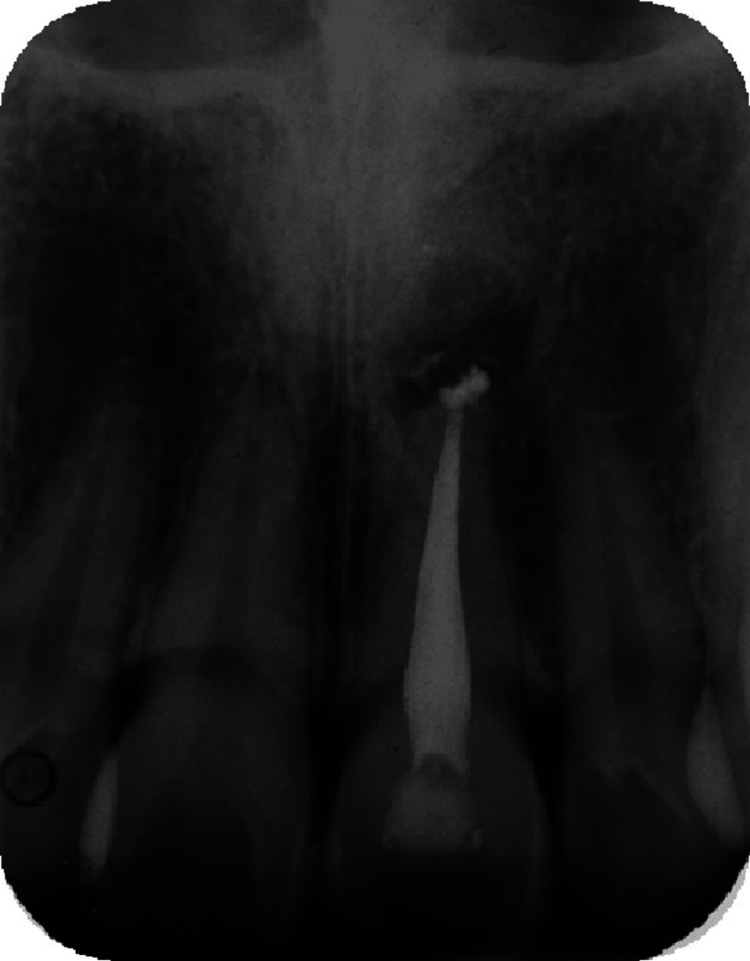
Preoperative radiograph of tooth 21

Radiographic investigation revealed that tooth 21 had restoration extending into the pulp chamber, a well-compacted homogenous and tapering root filling extending to the full length of the canal. The apical radiolucency measured 4.0 mm x 4.5 mm. There was radiopaque root filling material extruded past the root apex (Figure 3).

Another investigation that was performed was crack detection using transillumination light. There were no light refractory changes, and only craze lines were detected on the enamel surface. Tooth 21 was diagnosed as previously treated, symptomatic apical periodontitis. Other findings include fractured incisal edges on teeth 11 and 21. There was also localized plaque-induced gingivitis. The aim of treatment was to eliminate bacteria and infection in the root canal system of tooth 21 to encourage periapical tissue healing. As the patient was concerned about the discoloration, another aim was to restore the esthetics of tooth 21.

The treatment plan was full mouth scaling and prophylaxis, composite restoration on 11, and non-surgical root canal retreatment on tooth 21. If symptoms of tenderness on biting persist, endodontic microsurgery was planned for tooth 21. The course of treatment took a year and a half to complete.

After completion of oral hygiene instructions, scaling, and polishing, non-surgical root canal retreatment on tooth 21 was carried out. Upon administration of local anesthesia with 2% mepivacaine hydrochloride and placement of the dental dam, an access cavity was made through the palatal surface to reach the pulp chamber. The root filling material was identified as gutta-percha and was removed using ProTaper Rotary Retreatment files size D1, D2, and D3 (Dentsply Maillefer, Ballagues, Switzerland). Remnants of gutta-percha were dissolved with chloroform and paper points which had excellent wicking properties.

**Figure 4:**
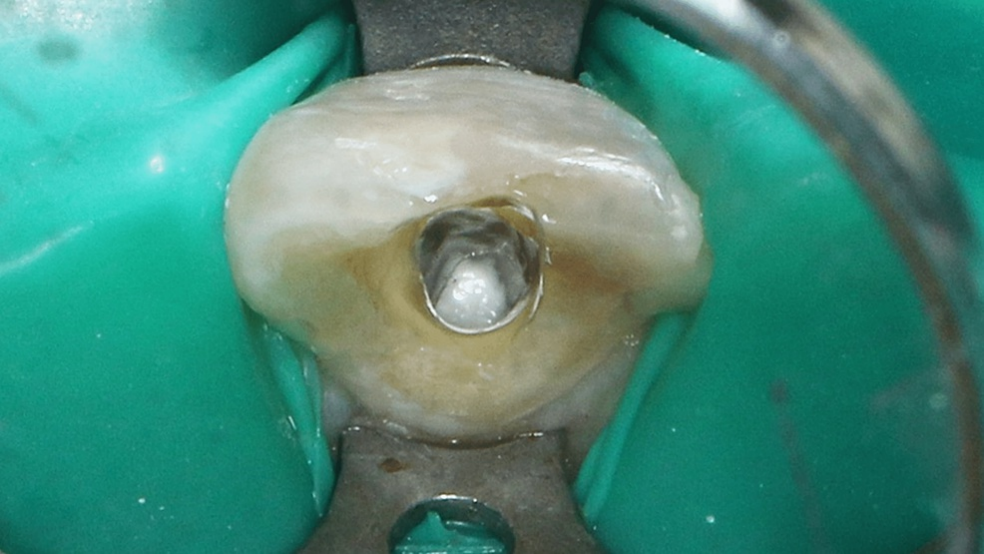
Intraoral picture of MTA placement on tooth 21

**Figure 5:**
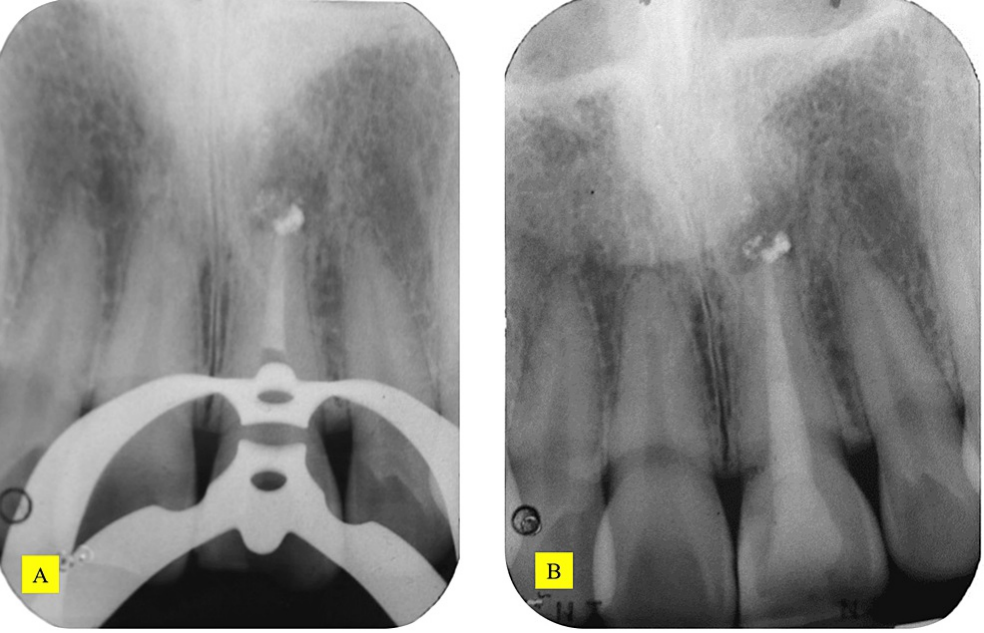
MTA placement radiograph and post-obturation radiograph of tooth 21 (A) MTA placement radiograph. (B) post-obturation radiograph

Working length was determined using an electronic apex locator (Root ZX Mini, J.Morita, Tokyo Japan) and was verified with a periapical radiograph. The corrected working length was 21.5 mm. The canal was irrigated with 2.5% sodium hypochlorite which had excellent bactericidal and tissue-dissolving properties. The canal was prepared with Protaper Next Rotary X1, X2, and X3 files and K-files size 10 to 30 (Dentsply Maillefer, Ballagues, Switzerland). Calcium hydroxide Calcipex II (Nishika, Shimonoseki City, Japan) was placed, and the access cavity was sealed with Cavit (3M Espe, Germany) and IRM (Dentsply Caulk, United States). The canal was irrigated with 2.5% sodium hypochlorite, 17% ethylenediamine tetraacetic acid, saline, and 2% chlorhexidine. Subsequently, the canal was dried with paper points. As the master apical size was 60, mineral trioxide aggregate (MTA) (Pro Root MTA, Dentsply Tulsa Dental Specialties, Tulsa, OK, USA) was placed incrementally as the apical seal (Figure 4). The apical barrier was measured at 5 mm. A radiograph was taken to ensure proper MTA placement. The rest of the canal was obturated using a thermoplasticized gutta-percha using the warm vertical compaction method (Figure 5). The tooth was then restored with composite shade A3 Filtek ᴵᴹZ250 XT (3M ESPE, Minnesota, USA) according to standard bonding protocol. An obturation radiograph was obtained.

**Figure 6:**
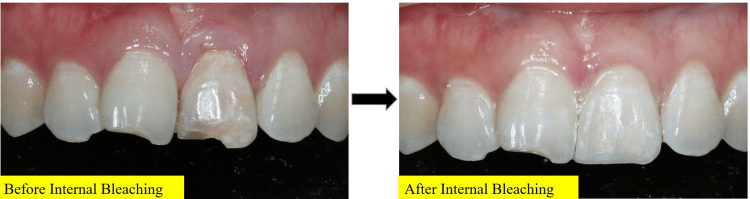
Before and after two cycles of internal bleaching on tooth 21

As the tooth was discolored, two cycles of internal bleaching using 35% hydrogen peroxide (Opalescence Ultradent, Utah USA) were completed. The process of internal bleaching includes steps of placing the bleaching agent in the pulp chamber and a temporary restoration over the pulp chamber. This walking bleaching technique allows the hydrogen peroxide to break down pigment molecules embedded in the dentinal tubules and enamel matrix for 7-10 days. The shade improved from B4 to A2. Once the patient was satisfied with the shade, composite restoration shade A2 (Filtek ᴵᴹZ250 XT (3M ESPE, Minnesota, USA) was placed (Figure 6). At this stage, the patient still felt sensitive on percussion and palpation with a score of 4/10.

**Figure 7:**
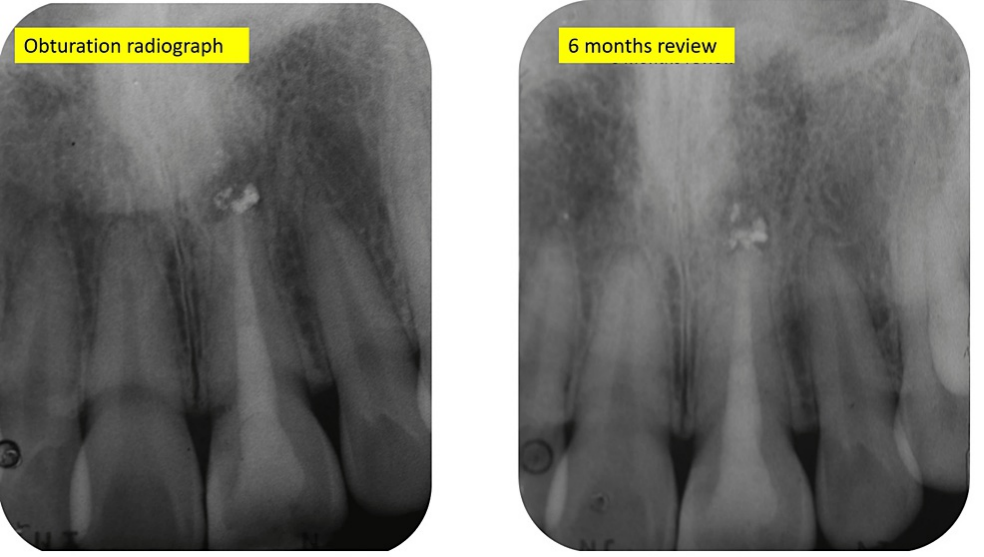
Postoperative radiograph and six-month review radiograph of tooth 21

**Figure 8:**
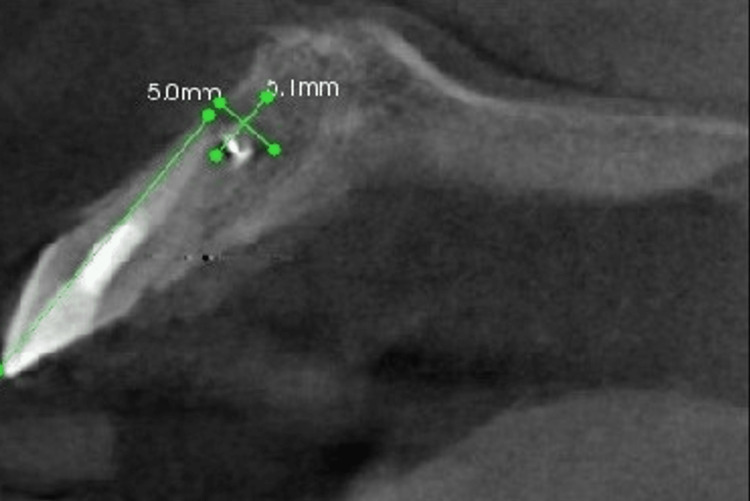
CBCT of tooth 21

At six months of follow-up, the patient still complained of having sharp pain on biting. Examination revealed sharp pain on percussion. To explore further, a periapical radiograph and limited-view CBCT (Planmeca ProMax® 3D Classic) were taken (Figures 7, 8). From the radiographs, the apical radiolucency remained. The foreign radiopaque material around the radiographic apex remained the same as in the preoperative radiograph. The radiopaque material was located at the base of the bony crypt. The width and depth of the lesion were 5.1 mm by 5.0 mm based on the CBCT measurement.

Therefore, endodontic microsurgery of Tooth 21 was carried out. For preoperative pain control measures, Ibuprofen 400 mg was given to the patient orally 30 minutes before the surgery. Mepivacaine hydrochloride 2%, 1: 100 000 adrenaline was administered as nasopalatine nerve block and buccal infiltration at the mucolabial fold. Another cartridge of 4% articaine was administered as supplemental buccal infiltration.

**Figure 9:**
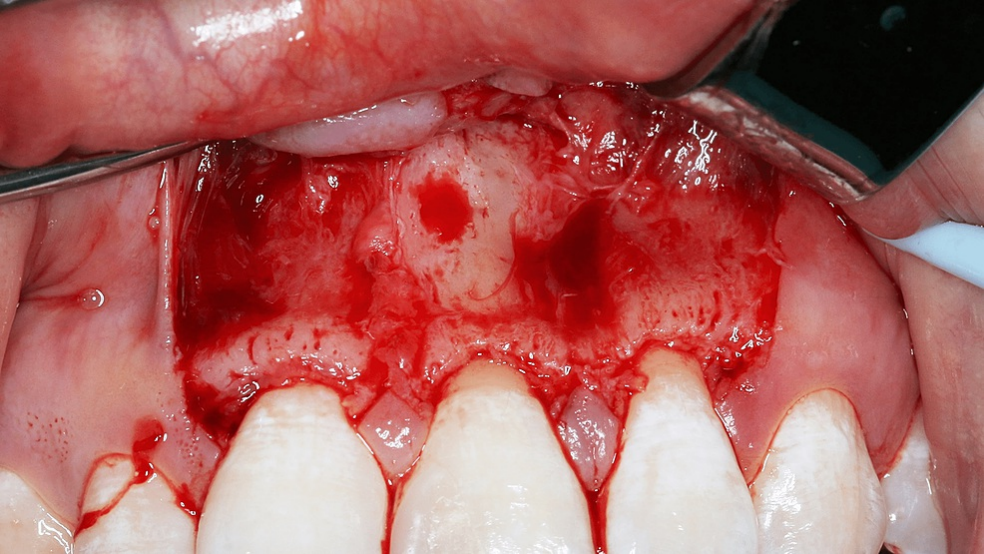
Flap design: Velvart's papilla-based incision

The choice of flap design was a full mucoperiosteal flap with Velvart’s papilla-based incision design. The gingiva and mucosa were incised using a 15C blade. At the sulcus and base of the papilla, a partial thickness flap was raised followed by a full mucoperiosteal flap raised onwards to the mucosa. A flap with two vertical incisions was made extending from the distal of tooth 11 to the distal of tooth 22 (Figure 9).

**Figure 10:**
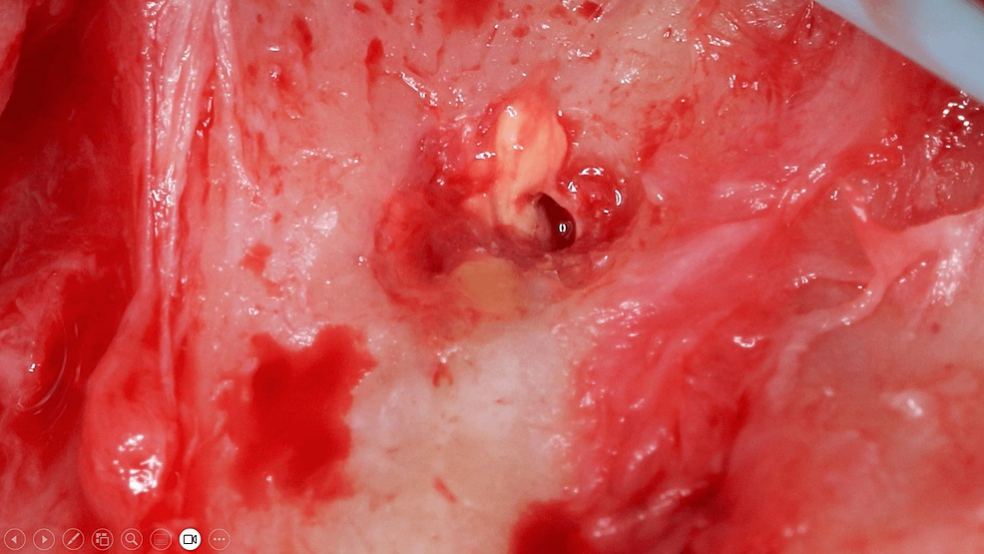
Sloughing of the gutta-percha at the periapical area

Based on the CBCT measurement, the distance from the alveolar crest to the foreign material was measured. The location of the lesion was estimated using the aid of a periodontal probe. The foreign body material located in the bony crypt was semi-solid, orange in color, and sloughing from the bony crypt (Figure 10). The material was identified as gutta-percha and was removed.

**Figure 11:**
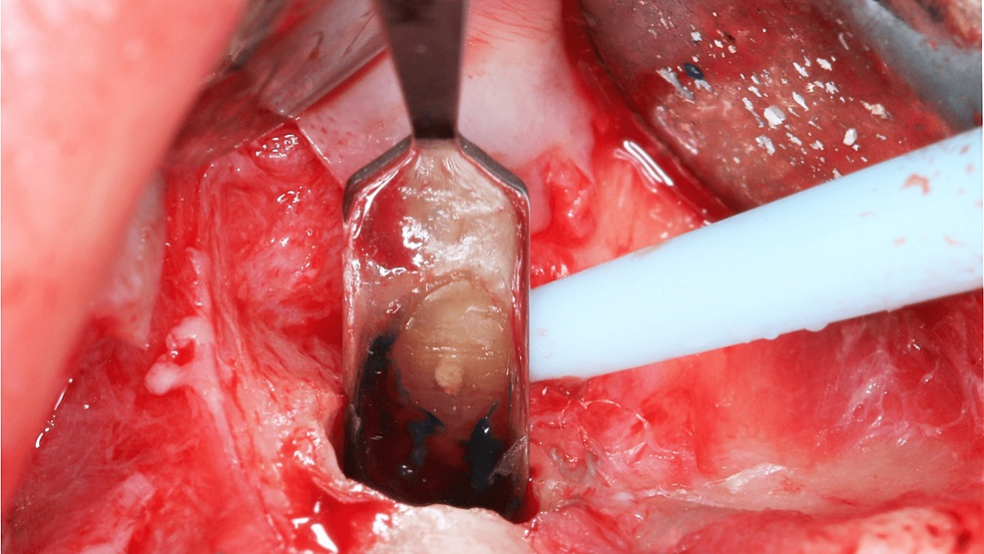
Apical root resection and investigation of cracks with methylene blue dye

**Figure 12:**
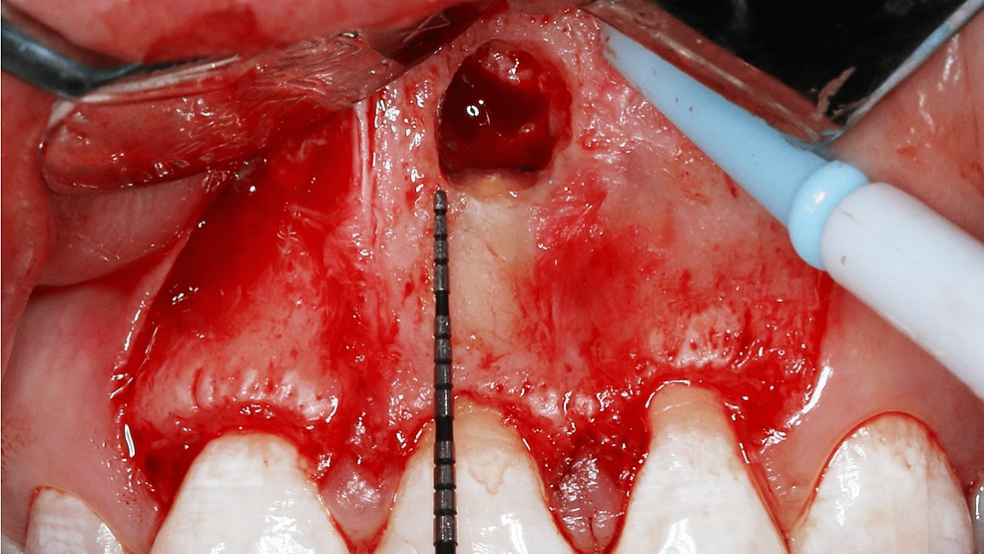
Remaining facial alveolar bone height

**Figure 13:**
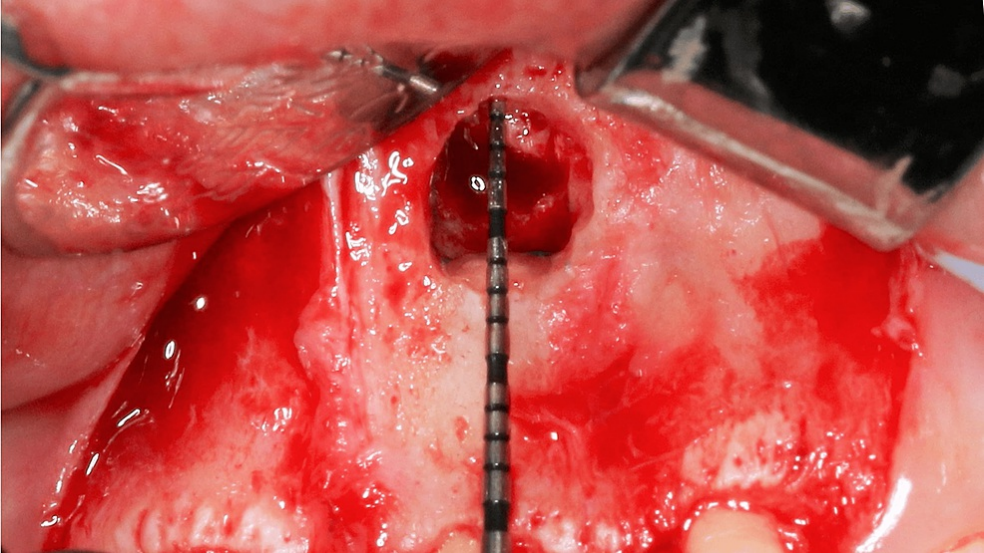
Height of the bony crypt

**Figure 14:**
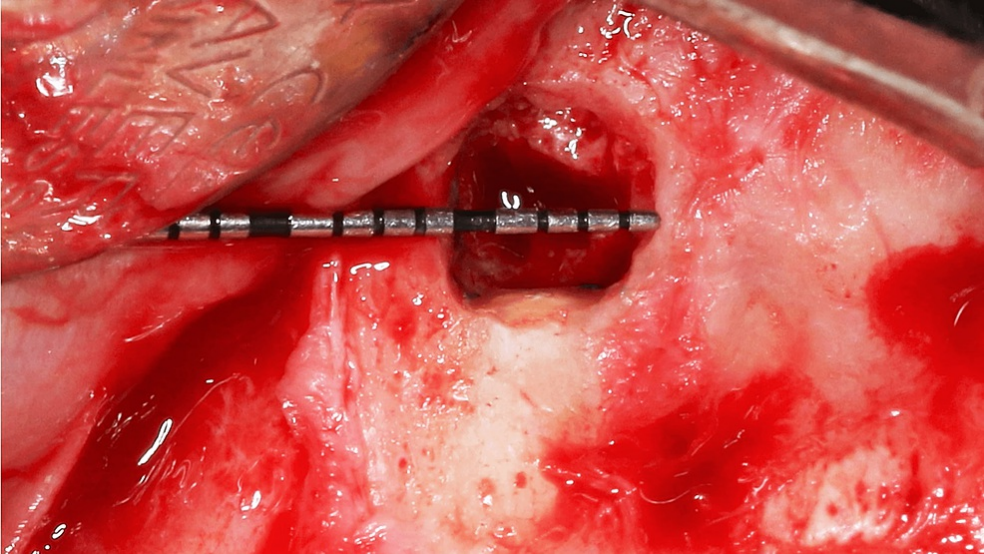
Width of the bony crypt

Any remaining debris was rinsed with normal saline, and the sharp edges of the bony crypt were smoothened. Once the extruded gutta-percha was removed, 3 mm of the root was resected using Lindemann bur and impact 45 degrees handpiece. Methylene blue dye was applied to the resected surface to investigate cracks. The dye did not reveal any fracture lines (Figure 11). The height, width, and depth of the bony crypt were 6.5 mm, 5.0 mm, and 3.0 mm, respectively (Figures 12, 13, 14).

**Figure 15:**
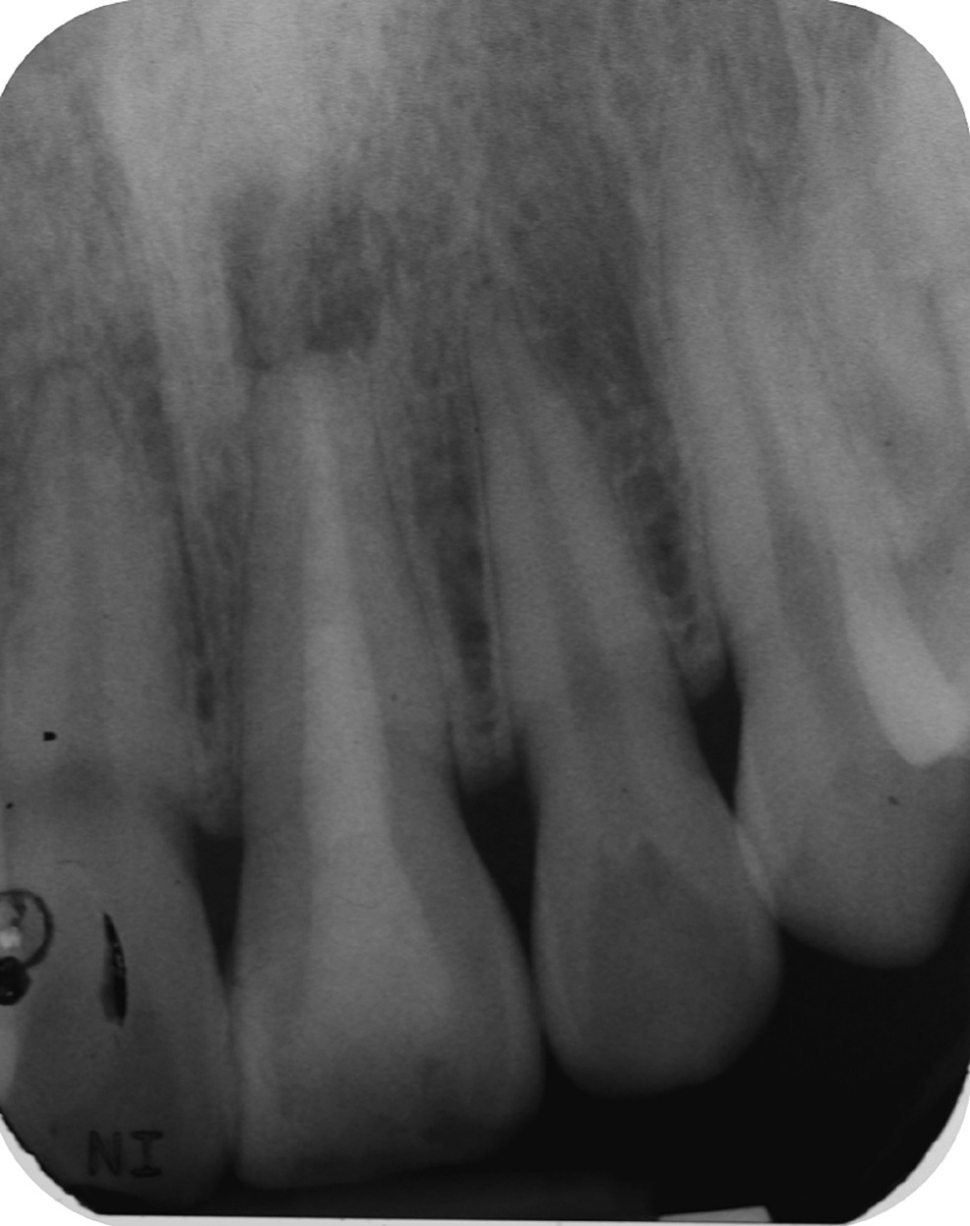
Radiograph after apical root resection

Subsequently, the surgical area was cleaned with saline, and the flap was approximated and sutured using Silk 5.0 suture. All endodontic surgical procedure was conducted under a dental operating microscope (Zeiss OPMI Pico, Germany). A postoperative radiograph was taken (Figure 15). Postoperative instructions on oral hygiene measures of the surgical site and dietary intake were advised.

**Figure 16:**
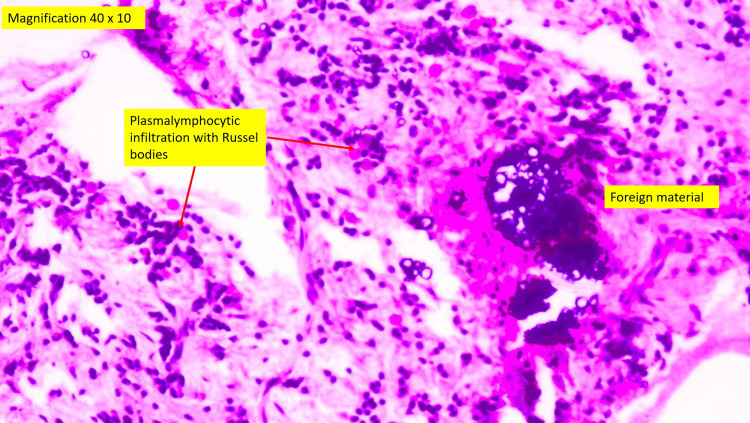
Magnification 400x of the hematoxylin and eosin staining

The specimen was sent for histopathological assessment. Hematoxylin and eosin staining showed the presence of fragments of dark foreign materials surrounded by inflamed fibrous connective tissue which consisted of plasma lymphocytic infiltration, a few Russel bodies, small vessels, and vital bone trabeculae (Figure 16).

**Figure 17:**
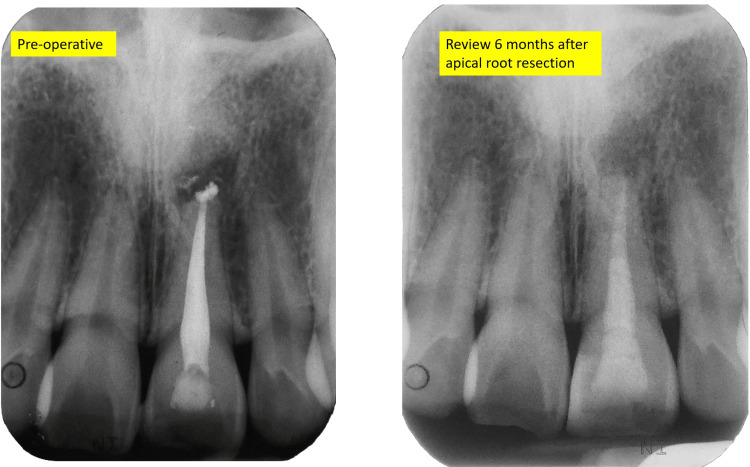
Periapical radiograph during six months of the review showing complete healing

The patient was reviewed three days later for suture removal. The healing was encouraging, although swelling was still present. During the six-month follow-up, the patient reported comfort and resolution of pain. A periapical radiograph was taken, and complete healing was shown by the presence of the re-establishment of the lamina dura over the resected surface. There was a complete bone repair. However, the density of the bone in the surgical site is dissimilar compared to the surrounding bone (Figure 17).

## Discussion

Persistent apical periodontitis is maintained by either intraradicular infection persisting in the apical root canal system [5], extraradicular infection, mostly in the form of periapical actinomycosis [6], foreign body reaction [7], and cystic lesions [8]. In this case, the presence of fine particles of gutta-percha in the periapical tissues evoked an intense, localized tissue response characterized by the presence of macrophages and giant cells. The accumulation of macrophages in conjunction with fine particles of gutta-percha caused impairment in the healing of apical periodontitis. Macrophages were known to release a battery of intercellular mediators which included pro-inflammatory cytokines and modulators that were involved in bone resorption.

Orthograde root canal retreatment and apical microsurgery when both performed, the success rate was higher at 81% than when either one treatment modality was performed separately [9]. Besides removing the apical third which consisted of 93% and 98% of lateral canals and apical ramifications respectively, the coronal and middle portions of the root canal system were thoroughly cleaned as well [10].

The buccal bone plate in this patient was intact and measured 10 mm. This is an important prognostic factor because the success rate with a buccal plate of more than 3 mm was higher at 94.3% [11]. The periosteum is believed to play a very important role as a source of immunocompetent cells and served as a barrier against epithelial cell migration into the healing sites.

The width of the bony crypt in this case was 5.0 mm. Smaller access window, an average of 7.04 mm in the mesio-distal direction healed significantly better than the mean access window of 8.60 mm [12]. In addition, the diameter of the radiographic lesion was 4 mm. The outcome was significantly higher with a success rate of 86% in teeth with radiographic lesions less than 5 mm compared with lesions of more than 5 mm which had a success rate of 65% [13].

There are conflicting studies on the size of periapical lesions in radiographs. A larger-sized periapical lesion measuring more than 5 mm preoperatively encourages the proliferation of fibroblasts from the periosteum into the bone defect which may result in scar formation instead of osseous regeneration after surgery [14]. This leads to a lower chance of healing. However, studies have also shown smaller lesions have better outcomes but are not statistically significant [15].

Although a wide range of retrofilling materials has been used, MTA has been shown to be superior compared to glass ionomer cement, SuperEBA, and amalgam with respect to better sealing ability, marginal adaptation, and biocompatibility [16]. Dye penetration studies, radioisotope, and bacterial penetration tests have demonstrated the presence of gaps and voids between retrofilling materials and the cavity wall. The best adaptation and least amount of gaps were found in cavities filled with MTA [17].

A papilla-based incision flap was chosen as the flap of choice as it has shown rapid healing and with no recession whereas complete mobilization of the papilla led to a marked loss of the papilla height. Especially in aesthetically crucial areas, papilla-based incision flaps avoid the opening of the interproximal spaces [18]. In the long-term, papilla-based incision flaps allow predictable recession-free healing of the interdental papilla as well. This is in contrast with the full mobilization of flaps which display a marked loss of the papilla height in the initial healing phase of one month [19] as well as one year postoperatively albeit less evident [20].

## Conclusions

Extrusion of obturation material can maintain the disease and interfere with post-treatment healing of apical periodontitis. Gutta-percha is considered biocompatible and well-tolerated by human tissues. However, in this case, the extrusion of gutta-percha evokes persistent postoperative pain and the lack of radiographic healing. Fine particles of gutta-percha evoke intense, localized tissue responses characterized by the presence of macrophages and giant cells. Therefore, extrusion of root filling material should be prevented to minimize postoperative pain and delayed of periapical healing as seen in this case. Endodontic microsurgery is an effective treatment for removing extruded root filling material with a very predictable outcome.
